# The Pho4 transcription factor mediates the response to arsenate and arsenite in *Candida albicans*

**DOI:** 10.3389/fmicb.2015.00118

**Published:** 2015-02-11

**Authors:** Verónica Urrialde, Daniel Prieto, Jesús Pla, Rebeca Alonso-Monge

**Affiliations:** Departamento de Microbiología II, Facultad de Farmacia, Universidad Complutense de MadridMadrid, Spain

**Keywords:** *Candida albicans*, stress response, transcription factor, oxidative stress, arsenate, arsenite, MAPK, signaling

## Abstract

Arsenate (As (V)) is the dominant form of the toxic metalloid arsenic (As). Microorganisms have consequently developed mechanisms to detoxify and tolerate this kind of compounds. In the present work, we have explored the arsenate sensing and signaling mechanisms in the pathogenic fungus *Candida albicans*. Although mutants impaired in the Hog1 or Mkc1-mediated pathways did not show significant sensitivity to this compound, both Hog1 and Mkc1 became phosphorylated upon addition of sodium arsenate to growing cells. Hog1 phosphorylation upon arsenate challenge was shown to be Ssk1-dependent. A screening designed for the identification of transcription factors involved in the arsenate response identified Pho4, a transcription factor of the myc-family, as *pho4* mutants were susceptible to As (V). The expression of *PHO4* was shortly induced in the presence of sodium arsenate in a Hog1-independent manner. Pho4 level affects Hog1 phosphorylation upon As (V) challenge, suggesting an indirect relationship between Pho4 activity and signaling in *C. albicans*. Pho4 also mediates the response to arsenite as revealed by the fact that *pho4* defective mutants are sensitive to arsenite and Pho4 becomes phosphorylated upon sodium arsenite addition. Arsenite also triggers Hog1 phosphorylation by a process that is, in this case, independent of the Ssk1 kinase. These results indicate that the HOG pathway mediates the response to arsenate and arsenite in *C. albicans* and that the Pho4 transcription factor can differentiate among As (III), As (V) and P_i_, triggering presumably specific responses.

## Introduction

Arsenic (As) is a metalloid widespread in nature. As occurs with others metalloids, it is toxic and the exposure to it is associated with a variety of diseases in humans, including immunological disorders (reviewed by Flora, 2011). Several compounds containing As are classified as human carcinogens according to the International Agency for Cancer Research. Arsenic can be found in the environment in two main forms: arsenate (As (V)) and arsenite (As (III)). Both prokaryotic and eukaryotic cells have developed mechanisms to take up and detoxify these compounds (Rosen, 2002), the oxidation state being essential for the type of transporter used in this process.

The budding yeast *Saccharomyces cerevisiae* has been considered as a trustworthy model to unravel the molecular details of metal action and their corresponding detoxification strategies (reviewed by Wysocki and Tamas, 2010). Arsenite enters the cell through the aquaglycerolporin, encoded by *FPS1* (Wysocki et al., 2001; Maciaszczyk-Dziubinska et al., 2010), in *S. cerevisiae* hexose permeases have been also involved in this process (Liu et al., 2004). Fps1 is a bidirectional channel than can also mediate the metalloid's efflux (Bienert et al., 2008). Arsenite is exported from the cytoplasm through the membrane Acr3/Arr3 transporters or transported into the vacuole as a glutathione-conjugated substrate by the Ycf1 ABC-transporter (Wysocki et al., 1997). The expression of these genes is regulated by the transcription factors Arr1 (also called Yap8) and Yap1. Yap1 also prevents oxidative damage in cells exposed to these compounds, by inducing target genes that remove ROS (Reactive Oxygen Species) generated by arsenic compounds (Menezes et al., 2008). Arsenate is a structural analog of inorganic phosphate (P_i_) and can easily enter the cell through phosphate transporters. Phosphate and arsenate can be imported into the fungal cell by Pho84 and Pho89 (two-high affinity permeases) and Pho87 and Pho90 (two low affinity permeases) (reviewed by Wysocki and Tamas, 2010). In the presence of arsenate, Arr2, an arsenate reductase, reduces arsenate to arsenite which is then removed from the cytoplasm either through Arr3 or Ycf1 (Tamas and Wysocki, 2001; Rosen, 2002).

In order to generate a detoxifying response, cells first have to sense the presence of toxic compounds. Signal transduction pathways mediated by MAP kinases are conserved mechanisms that allow cells sensing and responding to environmental stimuli (Kultz, 1998). These pathways included the MAPK module which is integrated by three protein kinases. These MAP kinases become sequentially activated by phosphorylation and control the expression of proper genes through different transcription factors. Signal enters MAPK module through transmembrane proteins, three-component system and/or other kinases. Sensing arsenite in *S. cerevisiae* is mediated, at least in part, by the HOG pathway (Sotelo and Rodriguez-Gabriel, 2006; Thorsen et al., 2006). The HOG pathway is integrated by two branches: the Sln1-Ypd1-Ssk1 that activates Ssk2 and Ssk22 and the Sho1-branch, which comprise Msb2, Sho1, Opy2, Hkr1 that triggers Ste11 phosphorylation. The MAPKKK Ssk2/Ssk22 and Ste11 phosphorylate the MAPKK Pbs2 which activates the MAPK Hog1. Hog1 is phosphorylated in response to arsenite (Sotelo and Rodriguez-Gabriel, 2006) among others stimuli, and induces a cell cycle G_1_-arrest in a Hog1-dependent manner (Migdal et al., 2008); moreover, it controls the transcription of genes involved in this response. Hog1 also modulates the arsenite uptake by Fps1 (Thorsen et al., 2006).

In addition to the HOG pathway, the cell wall integrity (CWI) pathway mediated by Slt2 is also involved in the response to arsenate (Matia-Gonzalez and Rodriguez-Gabriel, 2011). CWI pathway mediates the biogenesis of cell wall and incorporates different transmembrane proteins such as Cws1, Cws2, Cws3, Mig2, and Mtl1 which work as receptors (revised by Levin, 2011). These proteins trigger the MAPK module phosphorylation through different mechanisms. The MAPK module is integrated by: Bck1, Mkk1/Mkk2, and Slt2. Slt2 is phosphorylated upon arsenate addition and regulates the expression of several genes, many of them shared with the heat shock response. Arsenite and arsenate therefore trigger different cellular signals and, consequently, different responses in yeast.

Although *C. albicans* is not an environmental yeast, it was reported to be more resistant to different metal and metalloids than *S. cerevisiae* (Berdicevsky et al., 1993). The underlying mechanisms of this tolerance remain unknown. Nevertheless, similarly to *S. cerevisiae, C. albicans* also triggers Hog1 phosphorylation upon arsenite exposure and *hog1* mutants are more susceptible to this compound (Smith et al., 2004). This suggests that *C. albicans* and *S. cerevisiae* share a conserved response to arsenite although the structure of the ortholog pathway is different. In *C. albicans* the HOG pathway has a unique branch comprising a three-component system (Sln1-Ypd1-Ssk1) (**Figures 6B, 7**) that triggers the MAPK module which includes Ssk2, Pbs2, and Hog1. The transcription factor Sko1 has been reported to mediate osmotic stress response in a Hog1-depended way (Rauceo et al., 2008). To our knowledge, the signaling pathways involved in response to arsenate have not previously explored in *C. albicans*. In the present work we have investigated the signaling mechanisms involved in the response to arsenate, the dominant form of arsenic, in the opportunistic pathogen *C. albicans* and identified the transcription factor Pho4 as a mediator of the response to both arsenate and arsenite in this fungus.

## Materials and methods

### Strains and growth conditions

*C. albicans* strains used in the present work are listed in Table 1. *C. albicans* transcription factor mutant collection is deposited and available at the Fungal Genomic Stock Center (http://www.fgsc.net) (Vandeputte et al., 2012). The mutants used in this work are all homozygous null mutants in an Ura+ background unless otherwise stated. Yeast strains were grown in YPD medium (1% yeast extract, 2% peptone, 2% glucose), SD (Synthetic Dextrose, 2% glucose, 0.67% yeast nitrogen base without amino acids supplemented with synthetic complete dropout URA) or SD low phosphate (2% glucose, 0.57% yeast nitrogen base with ammonium sulfate, without phosphates, and without sodium chloride (MP Biomedicals) supplemented with synthetic complete dropout URA) supplemented or not with monobasic potassium phosphate (KH_2_PO_4_). Growth temperature was 37°C unless otherwise indicated. Growth in liquid medium was estimated as the absorbance at 600 nm (A_600_). Sensitivity on solid medium to different compounds was tested on plates supplemented with the indicated chemicals at the concentrations indicated in each assay; for these assays, cell number was adjusted to 10^8^ cells/ml and 1/10 serial cell suspensions were spotted and incubated overnight at 37°C. Wild type term refers to the corresponding non-mutated isogenic parental strain of the mutations under analysis; as mutants have different backgrounds, specific strain is indicated in the figures. As (V) or arsenate refers to Na_2_HAsO_4_(disodium hydrogen arsenate or sodium arsenate dibasic). As (III) refers to NaAsO_2_ or sodium arsenite. The media with arsenic compounds were correctly labeled and, then a specialized company was in charge of its discard.

**Table 1:** **Strains used in this study**.

**Strain**	**Genotype**	**Nomenclature in manuscript and figures**	**Source**
CAF2	*ura3Δ::imm434/URA3*	wt	Fonzi and Irwin, 1993
CAI-4	*ura3Δ::imm434/ura3Δ::imm434*		Fonzi and Irwin, 1993
RM100	*ura3Δ::imm434/ura3Δ::imm434 his1Δ::hisG/his1Δ::hisG-URA3-hisG*	wt	Negredo et al., 1997
CNC13	*ura3Δ::imm434/ura3Δ::imm434 his1Δ::hisG/his1Δ::hisG hog1::hisG-URA3-hisG/hog1::hisG*	*hog1*	San José et al., 1996
HI3-21	*ura3Δ::imm434/ura3Δ::imm434 hog1::hisG-URA3-hisG/hog1::hisG*	*hog1*	Prieto et al., 2014
VIC100	*ura3Δ::imm434/ura3Δ::imm434 his1Δ::hisG/his1Δ::hisG sko1Δ::hisG/sko1Δ::hisG-URA3-hisG*	*sko1*	Alonso-Monge et al., 2010
VIC200	*ura3Δ::imm434/ura3Δ::imm434 his1Δ::hisG/his1Δ::hisG hog1::hisG/hog1::hisG sko1Δ::hisG/sko1Δ::hisG-URA3-hisG*	*hog1 sko1*	Alonso-Monge et al., 2010
VIC100C	*ura3Δ::imm434/ura3Δ::imm434 sko1Δ::hisG/sko1Δ::hisG-URA3-hisG*	*sko1*	Alonso-Monge et al., 2010
CSSK21-1	*ura3::imm434/ura3::imm434 ssk1::hisG/ssk1::hisG–URA3–hisG*	*ssk1*	Calera et al., 2000
PVY121	*ura3Δ::λ imm434/ura3Δ::λ imm43 RPS1/rps1::[CIp10]*	wt	Vandeputte et al., 2012
BRY429	*ura3Δ::λ imm434/ura3Δ::λ imm43 rlm1::hisG/rlm1::hisG-URA3-hisG*	*rlm1*	Fungal Genetic Stock Center (http://www.fgsc.net)
CM1613	*ura3Δ::imm434/ura3Δ::imm434 mkc1::hisG/mkc1::hisG–URA3–hisG*	*mkc1*	Navarro-García et al., 1995
BEC63-19	*ura3::imm434/ura3::imm434 his1::hisG/his1::hisG hog1::hisG/hog1::hisG mkc1::hisG-URA3-hisG/mkc1::hisG*	*hog1 mkc1*	This work
JC482	*ssk2::loxP-ARG4-loxP/ssk2::loxP-HIS1-loxP*	*ssk2*	Cheetham et al., 2007
BRD3	*ura3Δ::imm434/ura3Δ::imm434 his1Δ::hisG/his1Δ::hisG pbs2Δ::cat/pbs2Δ::cat-URA3-cat*	*pbs2*	Arana et al., 2005
REP12	*ura3_::imm434/ura3_::imm434 ssk1::hisG/ssk1::hisG sho1::hisG/sho1::hisG-URA3-hisG*	*sho1 ssk1*	Román et al., 2005
CHO31-1	*ura3::imm434/ura3::imm434-URA3 ssk1::hisG/ssk1::hisG msb2::FTR/msb2::FRT sho1::hisG/sho1::hisG-URA3-hisG opy2::FRT/opy2::FRT*	*sho1 ssk1 msb2 opy2*	Herrero-de-Dios et al., 2014
SFY87	*ura3Δ::λ imm434/ura3Δ::λ imm434 his1::hisG/his1::hisG arg4::hisG/arg4::hisG RPS1/rps1::[CIp30]*	wt	Vandeputte et al., 2012
SFY5	*Mutant generated from BWP17 made by TIGR transposon collection*	*pho4*	Fungal Genetic Stock Center (http://www.fgsc.net)
SFY5-R	*pho4 ARD1/ard1::tTA P_tet-_PHO4myc-SAT1*	*PHO4^reint^*	This work
CAPL	*ura3Δ::imm434/ura3Δ::imm434 ADH1/adh1::prPHO4-CbLUC-URA3*	wt *PHO4 ^PR^-CbLUC*	This work
HOPL	*ura3Δ::imm434/ura3Δ::imm434 hog1::hisG/hog1::hisG ADH1/adh1::prPHO4-CbLUC-URA3*	*hog1 PHO4^PR^-CbLUC*	This work

### Molecular biology procedures and plasmid constructions

Standard molecular biology procedures were used for all genetic constructions. *C. albicans* was transformed using the lithium acetate method (Köhler et al., 1997). To reintegrate *PHO4* in the *C. albicans* genome, we constructed the pNIM1R-PHO4-myc plasmid as follows. The *PHO4* ORF was amplified using the primers: o-PHO4SalI-fwd (ACGCGTCGACATGGACCAGCAAGTTTGGAACCC) and o-PHO4NotI-rev (CCGCGGCCGCCCTTCCTTCCTTTCAACTCC). A 1.99 Kb fragment was amplified and subcloned in pGEM-T (Promega). Then, the *Sal*I-*Not*I fragment was accommodated in the *Sa*lI-*Not*I restriction of the pNIM1R-RFP plasmid (Prieto et al., 2014), generating the pNIM1R-PHO4-myc plasmid. This plasmid carries the *PHO4* ORF fused to myc under the control of a repressible tetracycline-regulated promoter. The pNIM1R-PHO4-myc plasmid was digested with *Ksp*I and *Apa*I for its integration in the *ADH1* genomic region of the *C. albicans* genome. The strain generated was designed as *PHO4*^reint^.

To quantify *PHO4* expression, we have used a *C. albicans* adapted version of the luciferase from Caribbean Click beetle *Pyrophorus plagiophthalamus* (CbLUC) (Accession Number KP202872) considering the codon usage of highly expressed genes (Prieto et al., 2014). A 3x HA epitope was placed at the C-terminal of CbLUC sequence as tag (Supplementary Figure 1). We constructed a luciferase plasmid to test promoter activity via the following steps: first, a *BamH*I-*Not*I fragment carrying the *URA3* marker from pCAHA plasmid (Peter Sudbery, University of Sheffield, UK) was ligated in a pDARD1 (Arana et al., 2007) replacing the *SAT1* marker gene and obtaining plasmid pDU0. This plasmid was modified to introduce an additional *Kpn*I site next to the 3′ARD1 integration sequence generating the plasmid pDUM0. The *Kpn*I restriction site was introduced amplifying the 3′ARD1 with o-3′-ARD1 Not (GGCCGCGGCCGCAAATAGCGTTCTATTGTCACCC) and o-3′ARD1 KpnI (GCTGGAGCTCGGTACCTAAAGATAGCAGCGACAAGGCC).

CbLUC-HA was first cloned into pNIM1R replacing the GFP ORF by digestion with *Sal*I and *Bgl*II restriction enzymes. The CbLUC with the *ACT1* terminator sequence was then obtained by PCR using the following primers: o-CbLUC XNS fwd (CCGCTCGAGGCTAGCAATAGTCGACGTGAATGGTTAAAAGAG) and o-ACTTerm S rev (GATACTAGTGGAATGAATGGGATGAATCATCAAAC). Then, the *Xho*I-*Spe*I fragment from the CbLUC-HA PCR was ligated with an *Xho*I-*Spe*I pDUM0 plasmid to generate pDUM0-L. Finally, the 5′upstream region of the PHO4 ORF was amplified using the following primers: O-PHO4pr fwd (GCTAGCGCTTGACAAAGTAATAAAGGTAAGC) and o-PHO4pr rev (GTCGACTGATTTTGCTGAAATCAATGTC). This 1.76 Kb fragment was subcloned in pGEM-T (Promega) and excised as a *Nhe*I-*Sal*I fragment which was then ligated in the *Nhe*I-*Sal*I sites of the pDUM0-L. The obtained plasmid, pDUM4-L was digested with *Kpn*I to direct its integration in the *ARD1 locus* in the *C. albicans* genome.

The *hog1 mkc1* mutant was generated by deleting the *MKC1* gene in a *hog1* mutant (CNC13) following the procedure previously described (Navarro-García et al., 1995). The major part of the coding sequence of the *MKC1* gene was replaced by the *hisG-URA3-hisG* cassette.

### Protein extracts and immunoblot analysis

Protein extracts were obtained from exponentially growing cultures (O.D. = 1) at 37°C in liquid medium after exposure to different compounds. Samples were taken at different time points and processed for western-blot as previously described (Martín et al., 1993). Equal amounts of proteins (assessed by 280 nm absorbance measurements) were loaded onto gels. Electrophoresis gels were performed at 10% polyacrylamide while electrophoretic shift was observed at 8% polyacrylamide gels. Blots were probed with the following antibodies: ScHog1 polyclonal antibody (Santa Cruz Biotechnology) that recognizes CaHog1 protein (43 kDa); Ab-p38-P (Thr180/Tyr182) 28B10 monoclonal antibody (Cell Signaling Technology, Inc.) which detects phosphorylated Hog1, anti-Mkc1 antibody (Navarro-García et al., 2005) that recognizes Mkc1 protein, Ab-phospho-p44/42 MAP kinase (Thr202/Tyr204) (Cell Signaling Technology, Inc.) that recognizes MKc1 (59 kDa) and Cek1 (49 kDa) phosphorylated forms and anti-myc Tag (clone 4A6 (Millipore)). Antibodies were used at 1:1000. A fluorescent system (LI-COR Biosciences) that relies on an infrared imaging system with fluorescent secondary antibodies was used to detect and quantify the signal.

### Luciferase assay

Cells carrying the CbLUC under the control of the *PHO4* promoter were grown in liquid medium at 37°C and exposed to the compounds under analysis. 10 ml samples were taken, centrifuged (5000 g × 5 min) to obtained cells, washed twice with distilled water, and then resuspended in CbLUC buffer (50 mM MgSO_4_, 30 mM sodium citrate pH 2.5) to a final concentration of 5 × 10^7^ cells/ml. For each determination, 50 μl of cell suspensions were mixed with the same volume of 100 mM D-luciferin in DMSO (SIGMA). Luminescence was measured in an OPTOCOMP Luminometer during 30 s in integration mode. Results were expressed as fold increase over the control, which was considered the signal detected before substrate addition.

### Statistical analysis

Statistical differences among two or more groups were calculated using two-way ANOVA. Data are expressed as the mean of at least three experiments ± standard deviation (SD).

## Results

### Arsenate triggers Hog1 and Mkc1 phosphorylation

**Figure 1:**
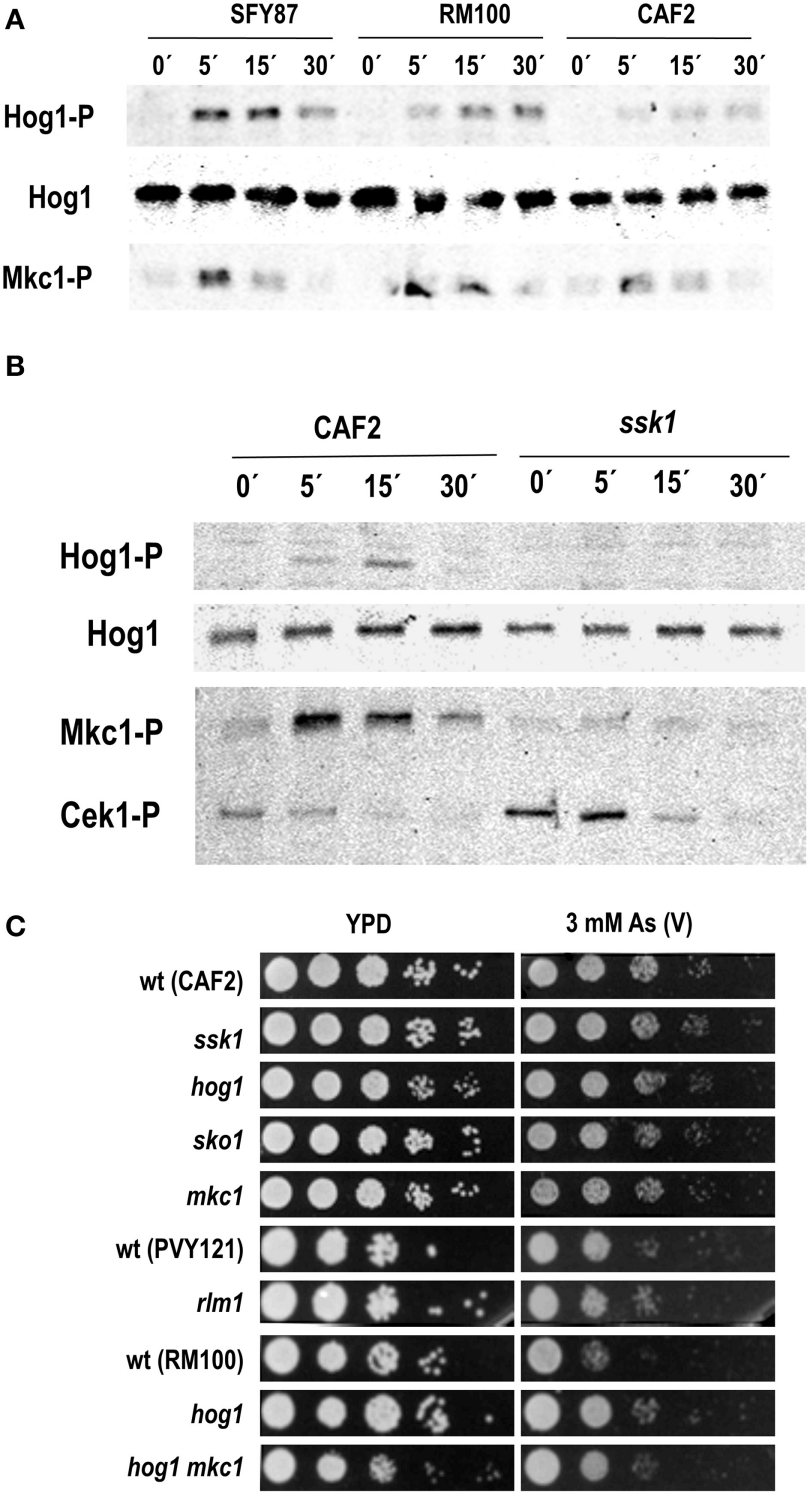
**Role of the MAP kinases in the response to arsenate. (A)** Three wild type *C. albicans* strains were challenged with 2 mM As (V) and samples collected at indicated time points. **(B)** CAF2 and *ssk1*-derived mutant were exposed to 2 mM As (V) and samples taken at the time indicated. MAPKs phosphorylation was detected using specific antibodies. Ab-anti Hog1 was used as loading control. Hog1-P, Cek1-P, and Mkc1-P indicate the phosphorylated form of the MAPKs. Hog1 indicates the total Hog1 protein. **(C)** Serial dilutions of the indicated *C. albicans* strains were spotted onto YPD plates (as a control) and YPD supplemented with 3 mM As (V). Mutant strains are shown above their parental strain. Plates were incubated at 37°C for 24 h.

In order to identify the MAPK pathway involved in arsenate signaling, different *C. albicans* wild type strains growing exponentially were challenged with 2 mM sodium arsenate and samples were analyzed at different time points. This compound triggered both Hog1 and Mkc1 phosphorylation which was detectable as early as 5 min after addition (Figure 1A). Hog1 phosphorylation was more prolonged in time than Mkc1 and lasted up to 30 min, while Mkc1 peaked at 5 min but returned to almost original levels at 30 min. Differences in Hog1 phosphorylation levels were observed among analyzed strains (Figure 1A): phosphorylation was more intense in SFY87 at short times compared to CAF2. Hog1 phosphorylation was fully dependent on Ssk1 as an *ssk1* mutant showed no Hog1 phosphorylation (Figure 1B). Mkc1 phosphorylation was also strongly reduced in the *ssk1* mutant, similarly to what has been described to occur in *hog1* and *pbs2* mutants (Arana et al., 2005) where Mkc1 H_2_O_2_-mediated signaling was highly dependent on the presence of active Hog1. The susceptibility to arsenate was tested on solid medium. The lack of Hog1 or Ssk1 did not lead to arsenate sensitivity on YPD plates supplemented with different As (V) concentrations (1, 2, and 3 mM arsenate were tested) (Figure 1C and data not shown) and this behavior was not dependent on the temperature of growth (data not shown). Remarkably, RM100 strain displayed an enhanced arsenate susceptibility compare to others wild type strains. Single *hog1* (as well as double *hog1 mkc1*) mutants displayed a susceptibility similar to other backgrounds mutants. The increased susceptibility to arsenate displayed by the RM100 strain was rescued in the presence of 1M sorbitol, an osmotic stabilizer that reverts some cell wall related phenotypes (data not shown). Additionally, mutants in the transcription factors Sko1 and Rlm1, known targets of the HOG and CWI pathways (respectively), showed no sensitivity to As (V). These results indicate that while the HOG and CWI pathways seem to be involved in the response to arsenate, their impairment do not cause an increased susceptibility to this compound.

### Screening of transcription factor mutants involved in the response to arsenate

With the aim of identifying the putative transcription factor involved in the resistance/tolerance to As (V), a 241 transcription factor knock out library from *C. albicans* was analyzed (available at Fungal Genetic Stock Center (http://www.fgsc.net)). Ten-fold serial dilutions of each strain were plated onto YPD plates supplemented with 1 mM or 2 mM arsenate. Five out of 241 mutants displayed susceptibility to arsenate compared to their isogenic parental strains. These genes are summarized in Table 2 while arsenate sensitivity is shown in Supplementary Figure 2. Among the mutants that displayed sensitivity to arsenate we identified *PHO4*, a basic helix-loop-helix (bHLH) transcription factor of the myc-family involved in the response to phosphate limitation. Since arsenate is structurally similar to phosphate, the *pho4* mutant was selected to perform further analyses.

**Table 2:** **Transcription factors whose deletion results in enhanced susceptibility to arsenate**.

**Gene name**	**Description CGD**	**Strain**	**Assembly 21 ORF number**
*PHO4*	bHLH transcription factor of the myc-family; required for growth in medium lacking phosphate and for resistance to copper and Phloxine B; induced by Mnl1 under weak acid stress	SFY5	orf19.1253
*ORF19.2260*	Putative transcription factor with zinc finger DNA-binding motif	CJN854	orf19.2260
*CAP1*	AP-1 bZIP transcription factor; apoptotic, oxidative stress response/Resistance, multidrug resistance; nuclear in oxidative stress; complements *S. cerevisiae yap1* mutant; oralpharyngeal candidasis-, human neutrophil, Spider biofilm induce	CJN608	orf19.1623
*WOR3*	Transcription factor; modulator of white-opaque switch; induced in opaque cells; promoter bound by Wor1; overexpression at 25° shifts cells to opaque state; deletion stabilizes opaque cells at higher temperatures; Spider biofilm induced	CJN432	orf19.467
*ADA2*	Zinc finger and homeodomain transcriptional co-activator; role in cell wall integrity and in sensitivity to caspofungin; required for the normal transcriptional response to caspofungin; required for yeast cell adherence to silicone substrate	CJN863	orf19.2331

### *In silico* analysis of *C. albicans* Pho4

The *PHO4* ortholog gene in *S. cerevisiae* encodes a basic helix-loop-helix (bHLH) transcription factor of the myc-family. This transcription factor activates transcription cooperatively with Pho2p in response to phosphate limitation being regulated by phosphorylation at multiple sites (Komeili and O'Shea, 1999). When environmental phosphate concentration is high, Pho4 is hyper-phosphorylated by Pho80-Pho85 complex, which switches off PHO pathway and excludes Pho4 from the nucleus. Excess of phosphate is stored in the form of poly phosphate in vacuoles (recently reviewed by Tomar and Sinha, 2014). When the environmental phosphate concentration is low, Pho81 represses the Pho80-Pho85 complex, leading to Pho4 hypo-phosphorylation. Then, Pho4 remains in the nucleus switching on the pathway and inducing the expression of high-affinity transporters (Pho84 and Pho89) and secretory phosphatases (Pho5, Pho11, and Pho12).

ScPho4 is a 312 aminoacid (aa) protein that becomes phosphorylated by the Pho80-Pho85 kinase complex on five Ser-Pro (SP) dipeptides (SP1, SP2, SP3, SP4, and SP6) (Komeili and O'Shea, 1999). Phosphorylation of SP2 and SP3 leads to the nuclear export of Pho4. Phosphorylation at SP4 site inhibits its nuclear import. Phosphorylation at SP6 blocks the interaction of Pho4 with the transcription factor Pho2. The function of SP1 phosphorylation has not been elucidated. Subsequently, unphosphorylated Pho4 is accumulated in the nucleus and controls the expression of phosphate-responsive genes. *CaPHO4* encodes a 659 aa length protein. The comparison between ScPho4 and CaPho4 shows that both proteins conserve a DNA binding motif (data base Pfam) at the C-terminal of the sequence (251–310 in *S. cerevisiae*, 595–648 in *C. albicans* (Supplementary Figure 3). Eight putative phosphorylatable Ser-Pro dipeptides can be detected in the CaPho4 sequence. Only one out of eight putative phosphorylation sites do concur when ScPho4 and CaPho4 are aligned. The presence of a Ser-Pro dipeptide together with other putative phosphorylation residues suggests that CaPho4 could be regulated by phosphorylation similarly to *S. cerevisiae* Pho4.

### Pho4 levels influence Hog1 phosphorylation on the presence and absence of stress

**Figure 2:**
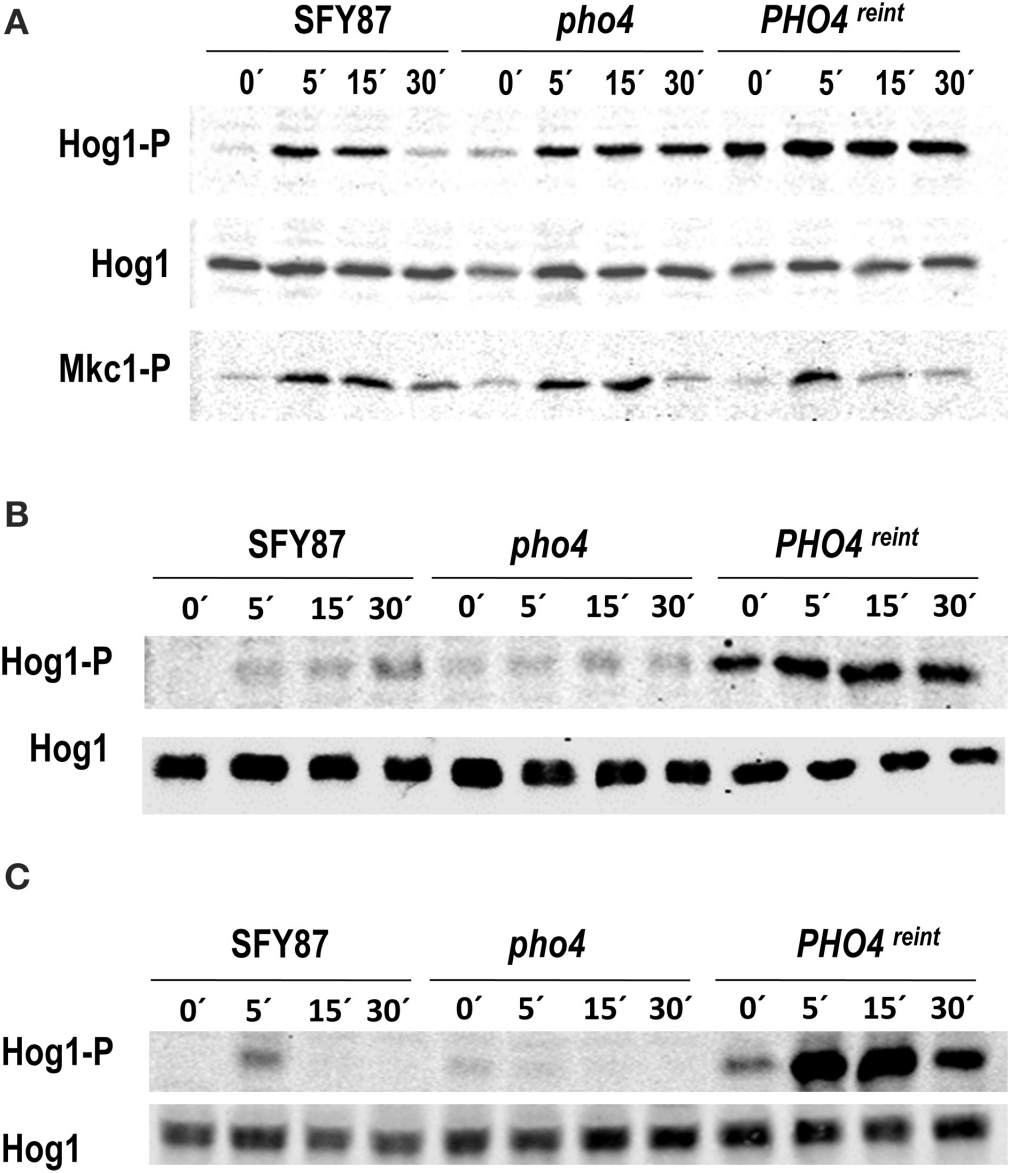
**Influence of Pho4 level in MAPK phosphorylation**. wt (SFY87), *pho4* mutant and *PHO4*^reint^ strains were challenged with **(A**) 2 mM As (V), **(B**) 1.5 M NaCl or **(C**) 5 mM H_2_O_2_. Samples were taken at different time points and processed for immunoblot. MAPK phosphorylated forms were detected using specific antibodies. Hog1-P and Mkc1-P designate the phosphorylated form of the MAPKs. Hog1 indicates the total Hog1 protein.

To allow Pho4 detection, the *PHO4* gene was epitope-tagged with myc and cloned in pNIM1R plasmid. This plasmid carries a repressible version of the doxycycline *OP4* promoter. The construction was integrated at the *ADH1* genomic region in the *pho4* mutant. The generated strain was named *pho4* reintegrant or *PHO4*^reint^. It must be remarked that in this strain, *PHO4* expression levels are constitutive and therefore, different to those derived from physiological levels. The reintegrant strain was included in the analyses and the role of Pho4 in MAPK phosphorylation was analyzed in the presence of As (V). The lack of *PHO4* did not impair Hog1 phosphorylation but rather, it prolonged it in time. Pho4 reintegration increased Hog1 phosphorylation under basal conditions (Figure 2A), that is, in the absence of arsenate. These data suggest that Pho4 levels do affect the response of Hog1 to arsenate. Mkc1 phosphorylation remained essentially unaffected by Pho4 (Figure 2A lower panel). We also checked the role of osmotic and oxidative stress (known to trigger Hog1 phosphorylation) in *pho4* mutant strains. Interestingly, the lack of Pho4 diminishes Hog1 phosphorylation upon 1.5 M NaCl or 10 mM H_2_O_2_ (Figures 2B,C). Conversely, Pho4 over-expression increased basal Hog1 phosphorylation and these cells were still able to increase Hog1 phosphorylation upon the addition of 1.5 M NaCl (Figure 2B) or 10 mM hydrogen peroxide (Figure 2C). These data indicated that expression of Pho4 is important for Hog1 signaling and supports a relationship between the HOG pathway and phosphate metabolism.

### Pho4 is involved in the response to arsenate

**Figure 3:**
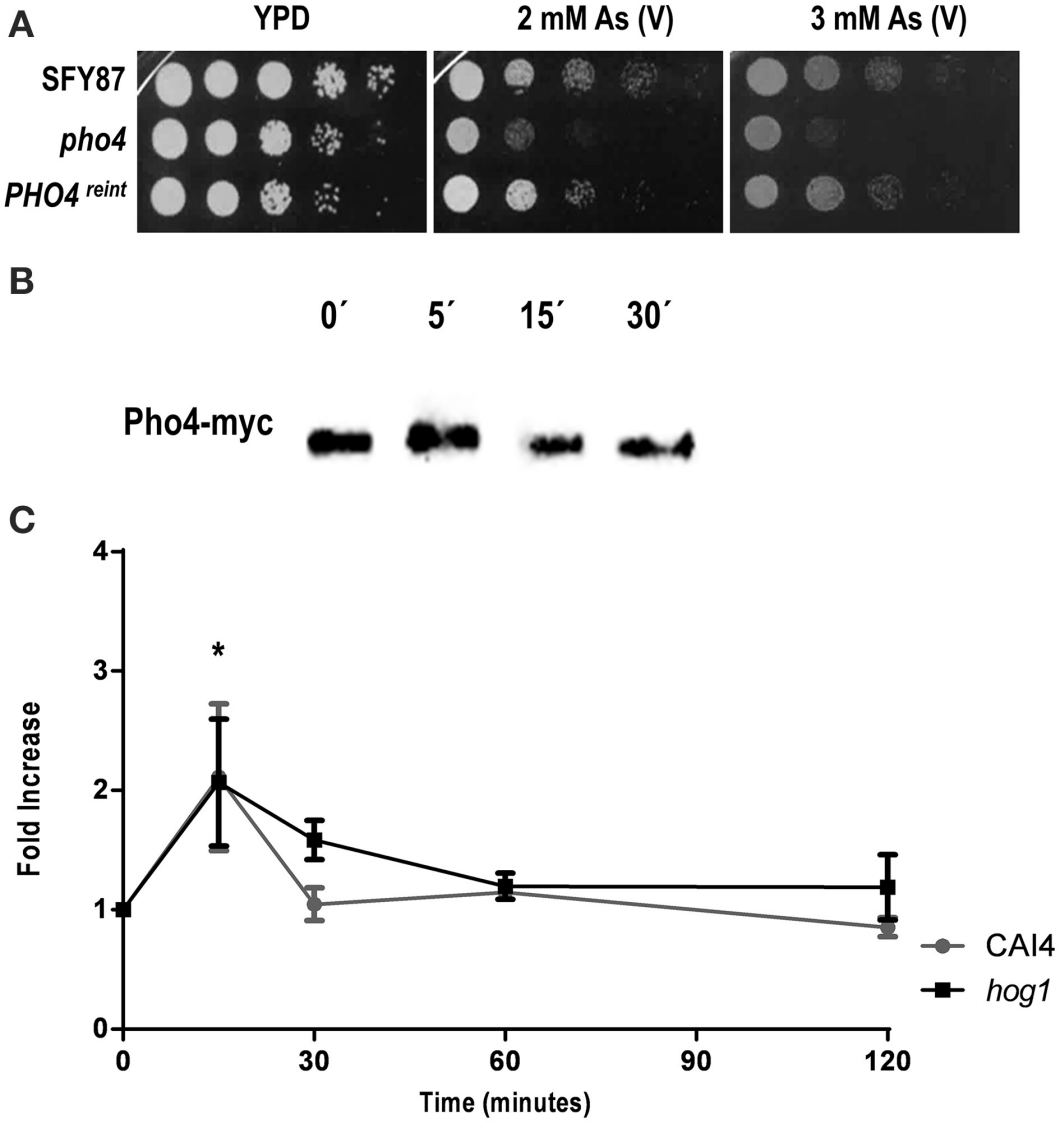
**Pho4 mediates the response to arsenate. (A)** Ten-folds cell dilutions of the indicated strains were spotted on YPD plates supplemented with 2 and 3 mM As (V). Plates were incubated at 37°C for 24 h. **(B)** Re-integrant *PHO4*-myc strain was challenged with 2 mM As (V) for different time points and samples collected and processed for immunoblot. Pho4-myc was detected using anti-myc antibodies. **(C)** Pho4 expression was quantified using *CbLUC* as gene reporter. CAI4 and *hog1* mutant strains carrying *PHO4^PR^*-*CbLUC* growing exponentially in YPD were challenged with 2 mM As (V) and samples were taken and processed for luminescence measurement. *PHO4* expression is expressed as fold induction related to YPD basal expression. Data represent the averages for three independent experiments. Error bars represent the standard deviation (SD). ^*^P ≤ 0.05.

The susceptibility to arsenate was tested between the parental wild type (SFY87) and both *pho4* and *PHO4*^reint^ mutants (Figure 3A). A *PHO4*^reint^ behaved as the wild type strain on As (V) supplemented plates, indicating that the *PHO4*-myc fusion was functional and that the susceptibility to arsenate was Pho4-dependent. The effect of As (V) on Pho4 was also analyzed. For this purpose, a *pho4* mutant carrying the ectopic *PHO4*-myc allele was challenged with 2 mM As (V) and samples were collected at different time points; Pho4-myc was detected using anti-myc antibodies (Figure 3B). A slight electrophoretic shift was detected within 5 min after As (V) challenge, indicating an increase in the molecular weight.

Pho4 expression was also quantified using a *C. albicans* codon optimized version of the Click beetle luciferase as gene reporter. For this purpose, a CAI4 wild type strain carrying the *PHO4^PR^*-CbLUC construction growing exponentially in YPD was challenged with 2 mM As (V). Pho4 expression was transiently and significantly induced 15 min after As (V) addition; this increase was independent on the presence of Hog1 as a *hog1* mutant displayed a similar pattern of Pho4 expression (Figure 3C). Collectively, these data indicate that As (V) induced both the expression of Pho4 expression and an electrophoretic delay, presumably due to Pho4 phosphorylation, while the lack of Pho4 rendered mutant sensitive to As (V). We therefore conclude that Pho4 mediates the response to arsenate.

### Pho4 differentiates between arsenate and phosphate

**Figure 4:**
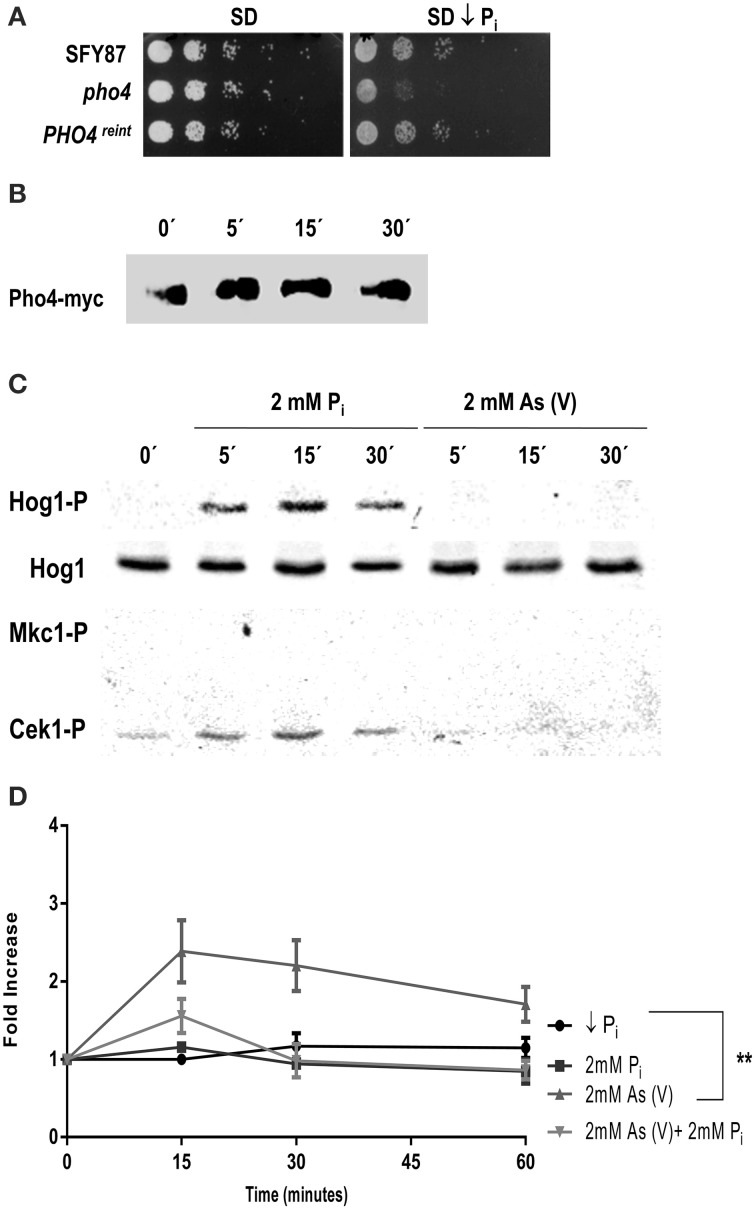
***C. albicans* differentiates between phosphate and arsenate. (A)** Growth of the indicated strains on SD and SD low phosphate plates. Plates were incubated at 37°C for 24 h. **(B)** *PHO4*^reint^ strain incubated in SD low phosphate overnight at 37°C was shifted to SD and samples were taken at different times points. Then, samples were processed for western-blot and Pho4-myc was detected using specific anti-myc antibodies. **(C)** The wild type strain CAF2 growing in SD low phosphate medium was challenged with 2 mM P_i_ or 2 mM As (V). Samples collected at different time points and MAPKs phosphorylation detected using specific antibodies. **(D)** The CAF2 strain carrying the *PHO4^PR^*-CbLUC grown exponentially in SD low phosphate supplemented with 2 mM P_i_ were washed twice and shift to SD low phosphate, SD low phosphate supplemented with 2 mM P_i_, 2 mM As (V) or 2 mM P_i_ plus 2 mM As (V). Samples were taken at time indicated and luminescence quantified. The graph represents the mean of four independent experiments. Error bars represent the standard deviation (SD). ^**^p ≤ 0.01.

The growth defect of *pho4* mutants on low phosphate SD medium plates was further analyzed. A drop test was performed comparing the growth on SD medium and SD medium prepared with a low phosphate Yeast Nitrogen Base (see Materials and Methods). *pho4* mutants displayed growth defects on low phosphate SD medium, indicating that Pho4 mediates adaptation to a low phosphate environment. As expected, the *PHO4*^reint^ was able to grow as the wild type strain (Figure 4A).

In low phosphate medium, *S. cerevisiae* Pho4 remains unphosphorylated but becomes phosphorylated when the phosphate concentration increases (reviewed by Tomar and Sinha, 2014). To analyze the behavior of Pho4 in *C. albicans*, the *PHO4*^reint^ strain was grown in SD low phosphate and shifted to SD supplemented with 2 mM inorganic phosphate (P_i_). Samples were taken at different time points and changes in the electrophoretic mobility analyzed (Figure 4B). A delay in mobility was observed after 5 min that lasted at least 15 min after the medium shift, suggesting that Pho4 became phosphorylated upon the increase of P_i_ concentration.

We also asked if P_i_ and As (V) were sensed similarly by the MAPK network due to their structural similarity. Cultures grown in SD low phosphate medium were split and challenged with either 2 mM P_i_ or 2 mM As (V). Phosphate triggered Hog1 and Cek1 phosphorylation while no phosphorylation was detected at all in response to arsenate (Figure 4C). These data indicate that *C. albicans* differentiates between arsenate and phosphate. Remarkably, arsenate is not sensed by MAP kinases in these experimental conditions. This effect was dependent on phosphate availability: when a similar experiment was performed starting from medium with phosphate (SD), the addition of P_i_ (2mM) or As (V) (2 mM) showed a complete different behavior: while P_i_ induced a slight activation of Hog1, As (V) triggered Hog1 and Mkc1 similarly to the results reported above (Figure 1 and Supplementary Figure 4).

The influence of phosphate in Pho4 expression was analyzed. For this purpose, we used cells from a CAI4 strain carrying the *PHO4^PR^*-CbLUC construction growing on SD low phosphate supplemented with 2 mM P_i_. Cells were collected, washed twice with SD low phosphate and released in different media: (1) SD low phosphate, (2) SD low phosphate plus 2 mM P_i_, (3) SD low phosphate plus 2 mM As (V) or (4) SD low phosphate plus 2 mM As (V) and 2 mM P_i_. Induction of Pho4 expression was followed on time (Figure 4D). The shift from low to 2 mM P_i_ supplemented medium did not have effect on Pho4 expression, at least at the time points analyzed. However, 2 mM As (V) led to a 2.5-fold induction of Pho4 expression at 15 min and decreased slightly remaining higher compare to the others conditions tested. When *C. albicans* cells were released on SD medium supplemented with equal amounts of arsenate and phosphate, the expression of Pho4 increased almost twice (similarly to the expression observed on YPD plus on 2 mM As (V) Figure 3C). Therefore, Pho4 increased its expression upon As (V) addition but not P_i_. This increase was higher when there was no phosphate in the medium. These results indicate that, despite their structural similarity, the cells sense differently arsenate and phosphate which, in turns, results in a different Pho4 expression.

### Pho4 is involved in the response to arsenite

**Figure 5:**
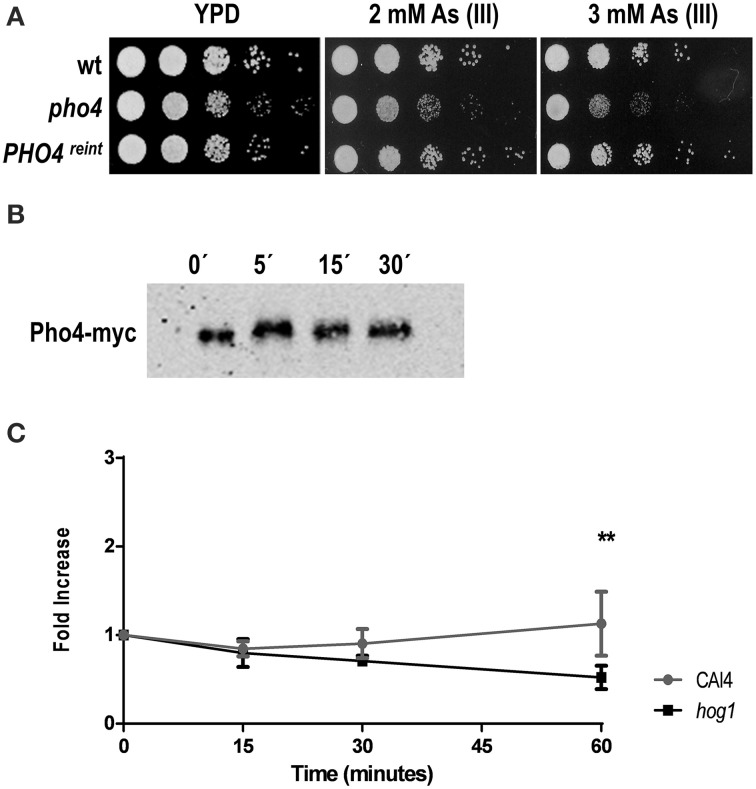
**Pho4 mediates the response to arsenite**. **(A)** Ten-fold cell dilutions of the indicated strains were spotted on YPD plates supplemented with 2 and 3 mM As (III). Plates were incubated at 37°C for 24 h. **(B)** *PHO4*-reintegrant strain was challenged with 2 mM As (III). Pho4-myc was detected using anti-myc antibodies. **(C)** The *PHO4* expression was quantified using the *PHO4^PR^*-CbLUC construction. CAF2 and *hog1* mutant carrying the gene reporter growing exponentially in YPD were challenge with 2 mM As (III). Samples were taken at time indicated and luminescence quantified. Graph represent the mean of three independent experiments and the error bars is the standard deviation (SD). ^**^p ≤ 0.01.

We also tested the role of Pho4 in the response to arsenite via drop test analyses performed on plates supplemented with 2 and 3 mM As (III). Similarly to the behavior on As (V) plates, the *pho4* mutant was sensitive to sodium arsenite, while *PHO4*^reint^ reverted the sensitive phenotype (Figure 5A). An electrophoretic shift was also observed in Pho4-myc mobility within 5 min after 2 mM As (III) addition (Figure 5B).

When the expression of Pho4 was analyzed upon 2 mM As (III) exposure, no induction of expression was detected 1 h after As (III) challenge in the wild type strain (Figure 5C). A statistically significant expression decrease was observed in the *hog1* mutant at 1 h of treatment. These data demonstrate that Pho4 is also involved in the response to arsenite, although differently compare to arsenate. *C. albicans* cells therefore, differentiate between As (V) and As (III) compounds.

### Arsenite triggers Hog1 phosphorylation in a Ssk1-independent way

**Figure 6:**
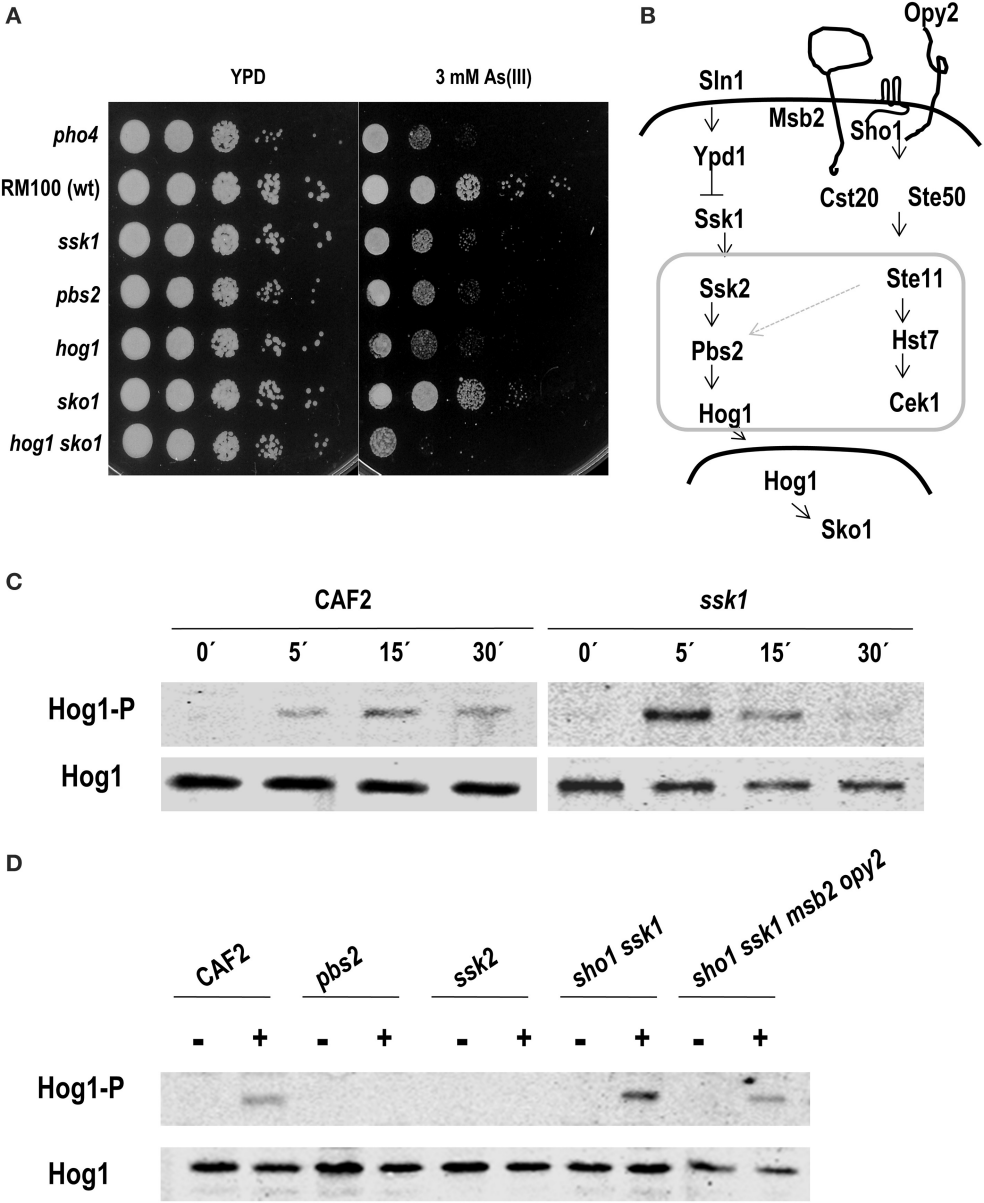
**Role of the HOG pathway in arsenite response**. **(A)** Drop test of the indicated strains on YPD plates supplemented with 3 mM As (III). Plates were incubated 24 h at 37°C. **(B)** Schematic graph of HOG and Cek1-mediated pathways in *C. albicans*. A possible connection between both pathways is indicated with a light gray arrow. MAPK modules are framed within a gray square. Different *C. albicans* strains were challenged with 2 mM As (III) at indicated time points **(C)** or at 10 min **(D)**. Hog1 phosphorylation was detected using anti-p38-P and total amount of protein was detected with anti-Hog1 antibody.

Lastly, we tested the role of the HOG pathway in the response to arsenite. Mutants defective in the HOG pathway were dropped on YPD plates supplemented with 3 mM As (III) and incubated at 37°C. Ssk1, Pbs2 and Hog1 defective mutants were sensitive to As (III) while no sensitivity was observed in the *sko1* mutant; a *hog1 sko1* double mutant was slightly more susceptible than single *hog1* mutant indicating a possible role for Sko1 in As (III) tolerance (Figure 6A). As (III) susceptibility of mutants defective in the CWI pathway were also tested, no significant differences were detected compare to the parental strain (Supplementary Figure 5). This fact suggests that HOG pathway may be relevant for a proper response to As (III). The ability of As (III) to trigger Hog1 phosphorylation was tested. Hog1 became phosphorylated within 5 min after sodium arsenite addition and lasted for 15–30 min (Figure 6C). Remarkably, Hog1 phosphorylation was detected in an *ssk1* mutant; although with a different kinetics, indicating that arsenite signaling is not fully Ssk1-dependent.

In order to determine the elements required for Hog1 phosphorylation upon arsenite addition, others signaling defective mutants were analyzed. We checked mutants lacking elements of the MAPK module (Pbs2 and Ssk2) and mutants lacking upstream elements of the HOG pathway in *C. albicans* (Ssk1). We also analyzed elements that mediate the HOG pathway in *S. cerevisiae* such as Sho1, Msb2, and Opy2 that in *C. albicans* participate in the Cek1 pathway (Figure 6B). Exponentially growing cells were exposed to 2 mM arsenite and samples were collected after 10 min (Figure 6D). Arsenite-induced-Hog1 phosphorylation depended on the MAPKK, Pbs2 and the MAPKKK, Ssk2. A *sho1 ssk1* double mutant (as well as the *sho1 ssk1 msb2 opy2* quadruple mutant) was able to trigger Hog1 phosphorylation similarly to wild type strain. Collectively, these data indicate that Hog1 phosphorylation upon arsenite challenge depends on the MAPK module of the pathway; however, the signal enters through an alternative way which is independent on Ssk1 and Sho1-Msb2-Opy2. *C. albicans*, therefore senses and responds via a different mechanism to arsenate and arsenite.

## Discussion

Eukaryotic cells have developed different mechanisms to sense and respond to environmental changes. Among these mechanisms, MAPK pathways are crucial since they participate in vegetative cell growth, mating and the response to different kinds of stress. MAPK pathways are integrated by a core of three MAP kinases that activate each other by sequential phosphorylation. Signal coming from upstream elements triggers MAP kinase phosphorylation which, in fact, controls the transcriptional expression of specific genes through defined transcription factors. These signal transduction pathways are well conserved in all eukaryotic organisms (Kultz, 1998) and allow cells to respond and adapt to environmental alterations. *C. albicans* responds to stress mainly through the HOG pathway, although it does so with the cooperation of others MAPKs routes (Monge et al., 2006; Román et al., 2007). Hog1 becomes phosphorylated is response to osmotic, oxidative and metal-induced stress and therefore, mutants defective in this pathway are sensitive to these kinds of stress (Alonso-Monge et al., 2003; Smith et al., 2004). Likewise, Hog1 becomes phosphorylated in response to As (III) and mutants in this pathway become sensitive to this compound.

**Figure 7:**
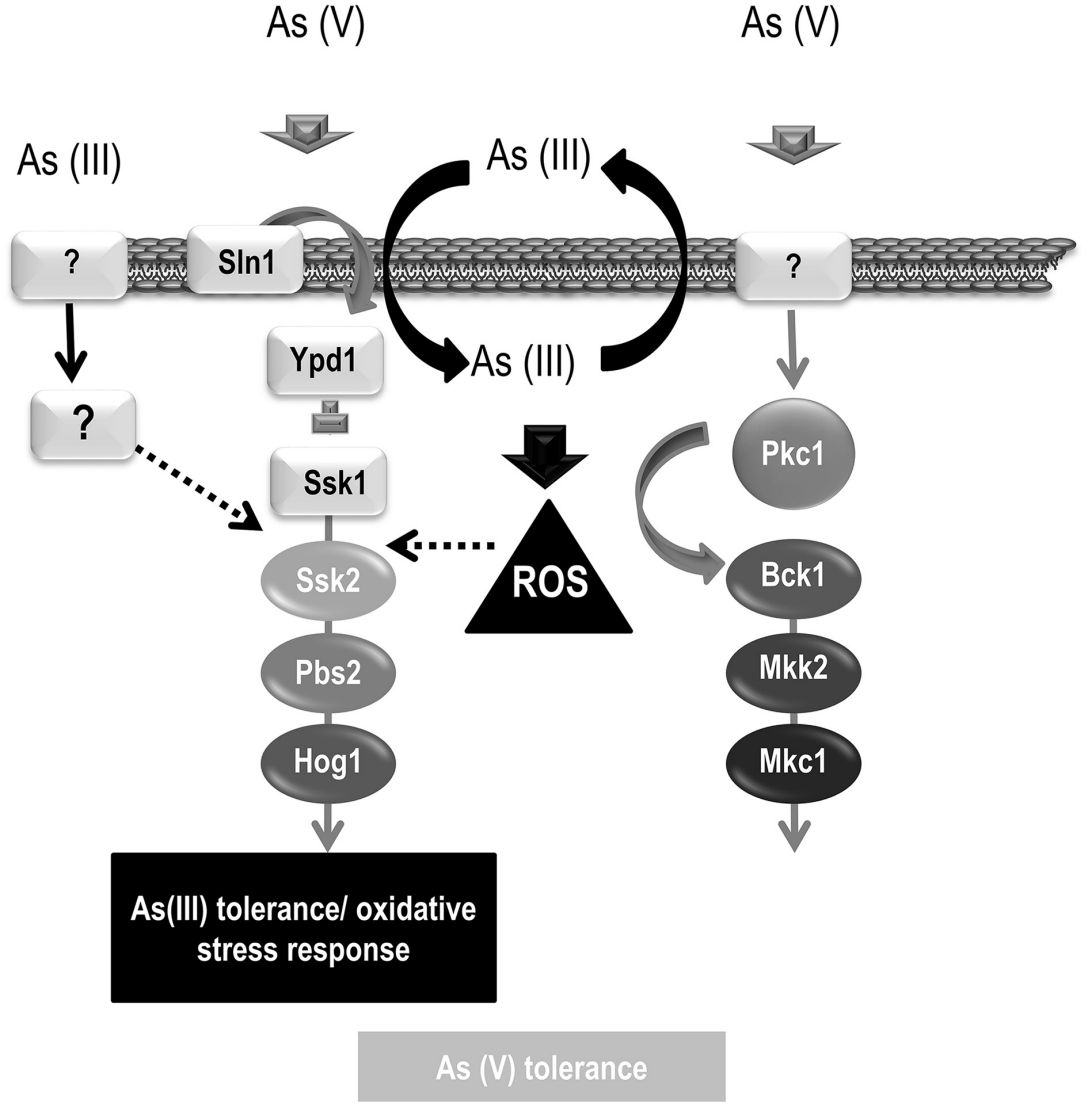
**Model of As (V) and As (III) signaling**. A schematic representation of the MAPK pathways involved in arsenic signaling is depicted. As (V) triggers phosphorylation of both, HOG and CWI pathways. As (V) signaling requires Ssk1 suggesting that signal comes from outside the cell. Hog1 and Mkc1 phosphorylation may mediate transcription of genes involved in its detoxification or tolerance. As (III) triggers Hog1 phosphorylation and therefore, HOG pathway may control the transcription of genes involved in As (III) detoxification. As (III) signaling is independent of Ssk1 and others elements involves in Cek1 phosphorylation (Sho1, Opy2, and Msb2) but requires Ssk2 and Pbs2. The As (III) signaling may enter through an unknown element (represented as a question mark) or may enter the cells triggering Hog1 phosphorylation avoiding the three components system (Sln1-Ypd1-Ssk1).

It has been reported that As (III) (the most toxic form of arsenic) induces ROS production in mammals (Liu et al., 2001) and yeast (Menezes et al., 2008). In *C. albicans* the production of ROS by As (III) has not been demonstrated. Nevertheless, As (III) signaling differs from oxidative stress signaling. In fact, Ssk1 has been shown to be necessary to trigger Hog1 upon hydrogen peroxide (Chauhan et al., 2003), supporting the view that *C. albicans* HOG pathway, unlike *S. cerevisiae*, integrates a unique signaling branch (Cheetham et al., 2007) (Figure 6B). Our data indicate that As (III) triggers Hog1 phosphorylation in an Ssk1-independent way. In *S. cerevisiae*, an Ssk1-independent Ssk2 activation upon osmotic challenge has been recently reported (Zhi et al., 2013). A similar alternative mechanism may explain Hog1 activation upon As (III) addition in *C. albicans*, since we observe Hog1 phosphorylation in *ssk1* but not in *ssk2* and *pbs2* mutants. Thus, Hog1 phosphorylation requires elements of the MAPK module, but the signal comes through an element different to Ssk1 (Figure 7). This putative element does not belong to the transmembrane proteins previously involved in Cek1 phosphorylation: Sho1, Msb2, and Opy2 (Herrero-de-Dios et al., 2014) (Figure 6B) whose homologs in *S. cerevisiae* also mediate Hog1 phosphorylation (Tatebayashi et al., 2007; Ekiel et al., 2009). Another possibility is that As (III), after its uptake in the cell, generates intracellular oxidative stress which, in turn, triggers Hog1 phosphorylation upstream Ssk2 in an Ssk1-independent way.

As (V) is sensed in a different way. In *S. cerevisiae*, As (V) triggers Slt2 (but not Hog1) phosphorylation (Matia-Gonzalez and Rodriguez-Gabriel, 2011). In this case, mutants in the CWI pathway are susceptible to arsenate while mutants defective in the HOG pathway are not (Matia-Gonzalez and Rodriguez-Gabriel, 2011). Unlike *S. cerevisiae*, As (V) induces Hog1 and Mkc1 phosphorylation in *C. albicans* but mutants in one or both pathways do not display enhanced susceptibility to arsenate on plate. The fact that CWI mutants do not show increased susceptibility to As (V) does not argue against its role in As (V) tolerance, as it has been already described that Mkc1 is activated by oxidative stress but *mkc1* cells are not sensitive to oxidant such as hydrogen peroxide (Navarro-García et al., 2005). Hog1 phosphorylation upon As (V) challenge requires Ssk1, suggesting that the signal is generated via the canonical membrane route, and presumably, outside to the cells (Figure 7). Ssk1 was also important to trigger Mkc1 phosphorylation in response to As (V); this key role played by the HOG pathway in controlling Mkc1 activation has been previously reported for hydrogen peroxide phosphorylation (Arana et al., 2005; Navarro-García et al., 2005) and demonstrate that MAPK pathways work coordinately. Interestingly, As (V) is only able to trigger MAPKs phosphorylation in rich medium (YPD) and neither Hog1 nor Mkc1 phosphorylation was detected when cultures were grown in phosphate deficient medium (Figure 4C). This different response could be explained because fungal cells express high-affinity phosphate transporters when phosphate is not available in surrounding environment. Phosphate starvation induces the phosphate signaling and response (PHO) pathway which controls the expression of specific genes such as the high-affinity phosphate transporter Pho84 (reviewed by Tomar and Sinha, 2014). This and others transporters/sensors with a higher affinity could be responsible for differentiating between phosphate and arsenate in spite of their structural similarity. In this phosphate starving environment, addition of inorganic phosphate triggered Hog1 and Cek1 phosphorylation. Cek1 become activated when cells resume growth from stationary phase to fresh medium (Román et al., 2005). Under our experimental conditions (a phosphate challenge in a phosphate deficient medium), Cek1 phosphorylation could be explained as a growth initiation signal after a starvation period. The expression of the high-affinity phosphate transporter Pho84 has been shown to be down-regulated in a *hog1* mutant in YPDAU medium (Enjalbert et al., 2006) indicating that the HOG pathway contributes, either directly or indirectly, to control its expression. Our work shows that Hog1 senses the presence of phosphate when environmental phosphate concentration is low. Therefore, it may contribute to phosphate homeostasis, presumably mediating Pho84 expression among others required proteins. The transcription factor involved in this regulation remains unknown and it is tempting to speculate that Pho4 could be a downstream Hog1-target. Our data do not demonstrate a direct connection between the HOG and PHO pathways, but suggest a relationship between both signaling mechanisms. A constitutive *PHO4* expression (*PHO4*^reint^) leads to an increased Hog1 basal phosphorylation while *pho4* mutants display a Hog1 altered phosphorylation pattern. These facts indicate that the intracellular concentration of Pho4 has to be tightly controlled, being otherwise a stress signal (phosphorylating Hog1) in *Candida* cells. On the other hand, the absence of Hog1 does not impair Pho4 expression upon As (V) challenge; but it does so upon As (III) addition.

Pho4 is required for growth in medium lacking phosphate in *C. albicans* (Figure 4A) (Homann et al., 2009; Romanowski et al., 2012). In *S. cerevisiae* Pho4 function depends on phosphate availability regulating its dynamic and phosphorylation (Komeili and O'Shea, 1999). When the phosphate concentration is high in the medium, Pho4 becomes hyper-phosphorylated by the Pho80-Pho85 complex which results in Pho4 being excluded from the nucleus; when the environmental phosphate concentration is low Pho81 represses the Pho80-Pho85 complex leading to Pho4 hypo-phosphorylation and Pho4 remains in the nucleus inducing the expression of high-affinity transporters (Pho84 and Pho89) and secretory phosphatases (Pho5, Pho11, and Pho12). A similar mechanism can be suggested for *C. albicans* since a delay in electrophoretic Pho4 mobility, probably due to Pho4 phosphorylation, was detected when a culture was shifted from a medium depleted in phosphate to normal phosphate availability.

In addition to the role of Pho4 in phosphate metabolism, it is also involved in As (V) tolerance in *C. albicans*. This role can be explained because As (V) is structurally homolog to inorganic phosphate (P_i_). As (V) also induces a delay in the electrophoretic mobility which suggests again phosphorylation. This putative phosphorylation(s) must switch off the transcription of high-affinity transporters and secretory phosphatases, thus avoiding the uptake of phosphate (or, in this case, environmental arsenate). It is also possible that Pho4 becomes phosphorylated at different sites upon Pi and As (V) addition, inducing specific and separated responses, since *C. albicans* can discriminate between Pi and As (V). This discrimination was observed in the signaling but also in Pho4 expression. As (V) induces Pho4 transcriptional activation (higher when environmental phosphate concentration is low) although phosphate starvation did not affect Pho4 expression (Figure 4B). This transcriptional induction is short in time and may correlate with the induction of an As (V) specific response.

Pho4 plays an additional role in As (III) tolerance. As (V) enters the cells through phosphate transporter and then, is reduced to As (III) by reductases. It is not surprising that mechanisms involved in As (V) tolerance could be implicated in As (III) adaptation as well. In this sense, Cap1 was identified in our screening searching for mutants sensitive to As (V). The homolog to Cap1 in *S. cerevisiae*, Yap1 controls As (III) response in cooperation with Met4 (Thorsen et al., 2007). Others transcription factors identified as implicated in As (V) tolerance in *C. albicans* were Ada2, Wor3, and orf19.2260. Zhou and co-workers showed that the *S. cerevisiae ada2* mutant was susceptible to arsenite in a genome-wide screening (Zhou et al., 2009). Our work, link these transcription factors to As (V) tolerance.

Why are mechanisms mediating As (III) and As (V) tolerance still functional in a non-environmental yeast? Although speculative, it is clear that humans are being constantly exposed to inorganic arsenic through food and drinking water causing different disorders (Flora, 2011). Intestinal microbiome plays an important role in arsenic detoxification in order to remove it from the body (Rowland, 1981; Lu et al., 2013). *C. albicans* belong to human gut microbiome and therefore it must promote As (and others chemicals) detoxification. Therefore, evolution may have well played a role in the maintenance and tuning of these mechanisms to cope with toxic arsenic compounds. In this context, coordinating MAPK signaling with the PHO pathways is an attractive mechanism that may be useful for adaptation to the commensal state.

## Conflict of interest statement

The authors declare that the research was conducted in the absence of any commercial or financial relationships that could be construed as a potential conflict of interest.

## Supplementary material

Supplementary Figure 1 **Sequences of *CbLUC* gene (A) and protein (B) adapted for *C. albicans***. Accession number: KP202872.

Supplementary Figure 2 **Sensitivity to arsenate displayed by transcription factor knock-out mutants**. Transcription factor mutants identified as sensitive to arsenate were spotted on YPD and YPD plus 2 mM arsenate and incubated at 37°C for 24 h.

Supplementary Figure 3 **Comparison of ScPho4 and CaPho4 sequences**. ScPho4 and CaPho4 were aligned using the default setting of Clustaw2. Dark arrows sign ScPho4 reported phosphorylation sites (Sp1, SP2, SP3, SP4, and SP6) while gray arrows indicate putative phosphorylation sites detected in CaPho4 sequence.

Supplementary Figure 4 **P_i_ and As (V) trigger different signaling pathways**. A wild type strain (CAF 2) grown exponentially in SD medium was split in two and 2 mM Pi or 2 mM As (V) was added to each culture. Samples were taken at different time points and MAP kinase phosphorylation was analyzed using specific antibodies. Hog1-P, Cek1-P, and Mkc1-P designate the phosphorylated form of the MAPKs while Hog1 indicates the total Hog1 protein.

Supplementary Figure 5 **Defective mutants in the CWI pathway are not susceptible to As (III)**. Ten-fold dilutions of the indicated strains were spotted on YPD and YPD plus 3 mM As (III). Plates were incubated at 37°C for 24 h.

## Abbreviations

As (V): arsenate as Na_2_HAsO_4_(sodium arsenate)
As (III): as NaAsO_2_ (sodium arsenite)
P_i_: inorganic phosphate.
