# Supplementary figures and images for: The Pho4 transcription factor mediates the response to arsenate and arsenite in Candida albicans
Source: Front Microbiol. 2015 Feb 11;6:118. doi: 10.3389/fmicb.2015.00118 (PMC4324303; doi:10.3389/fmicb.2015.00118)

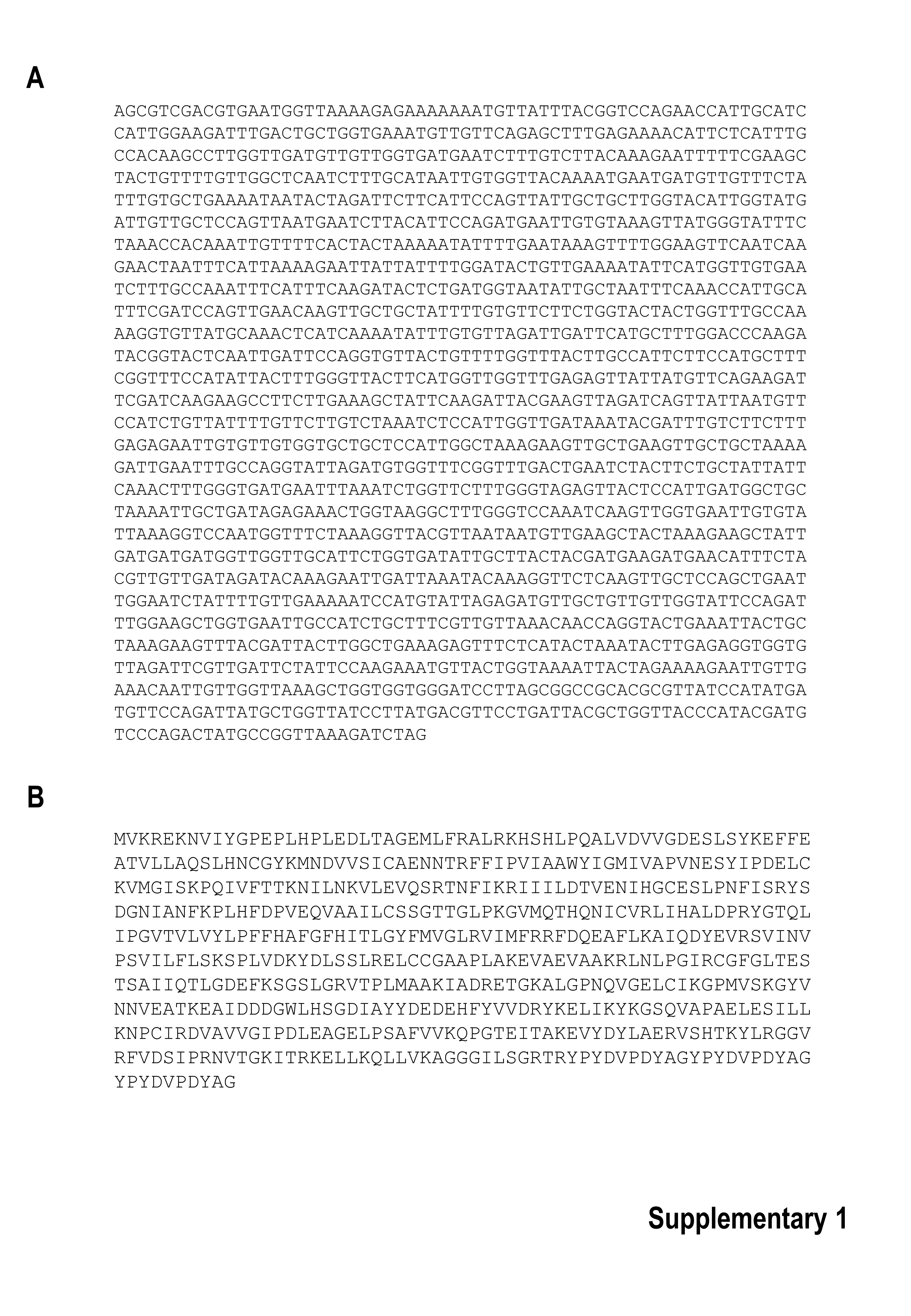

Supplement: Supplementary Figure 1 — Sequences of CbLUC gene (A) and protein (B) adapted for C. albicans. Accession number: KP202872. [file Image1.TIF]

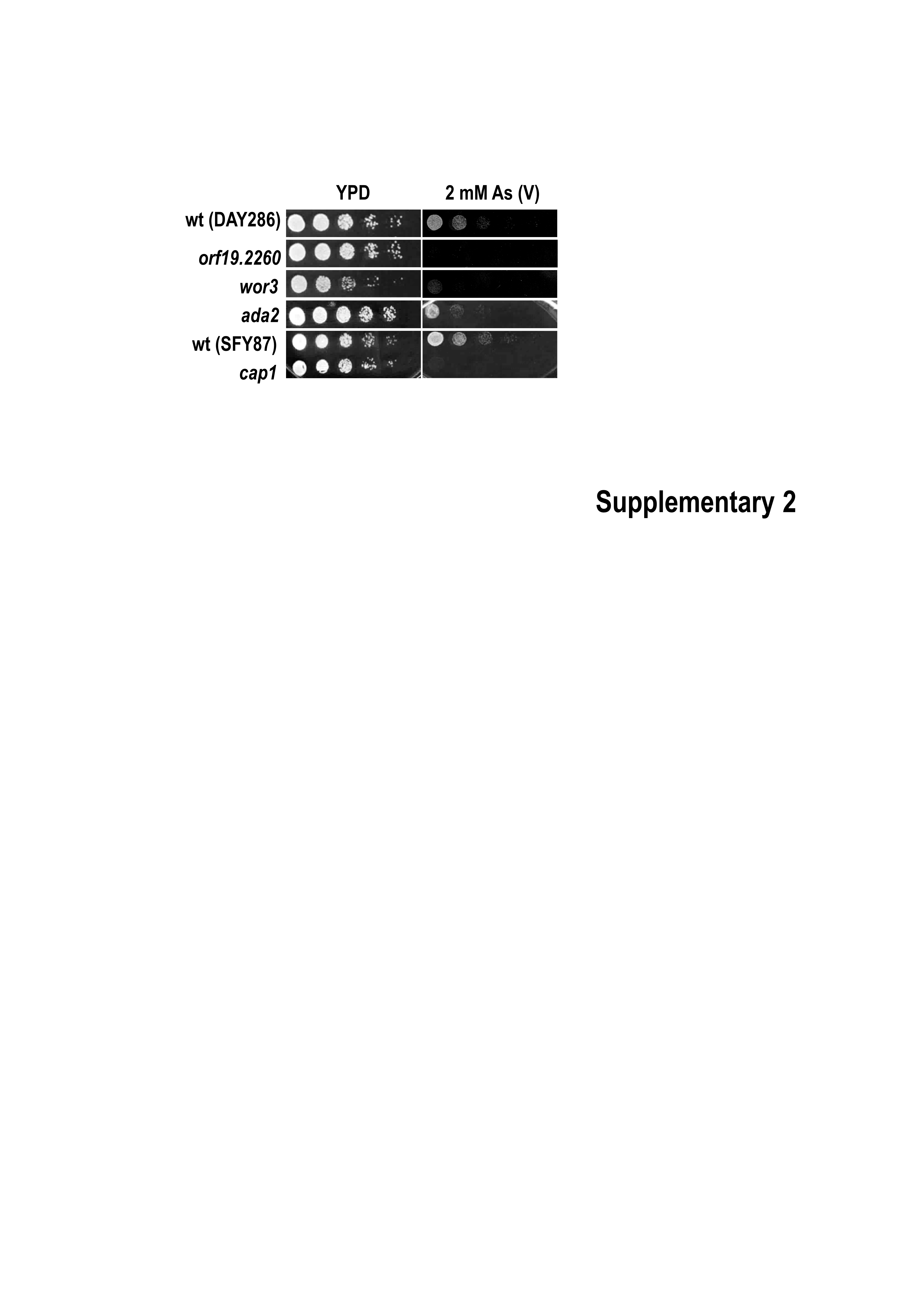

Supplement: Supplementary Figure 2 — Sensitivity to arsenate displayed by transcription factor knock-out mutants. Transcription factor mutants identified as sensitive to arsenate were spotted on YPD and YPD plus 2 mM arsenate and incubated at 37°C for 24 h. [file Image2.TIF]

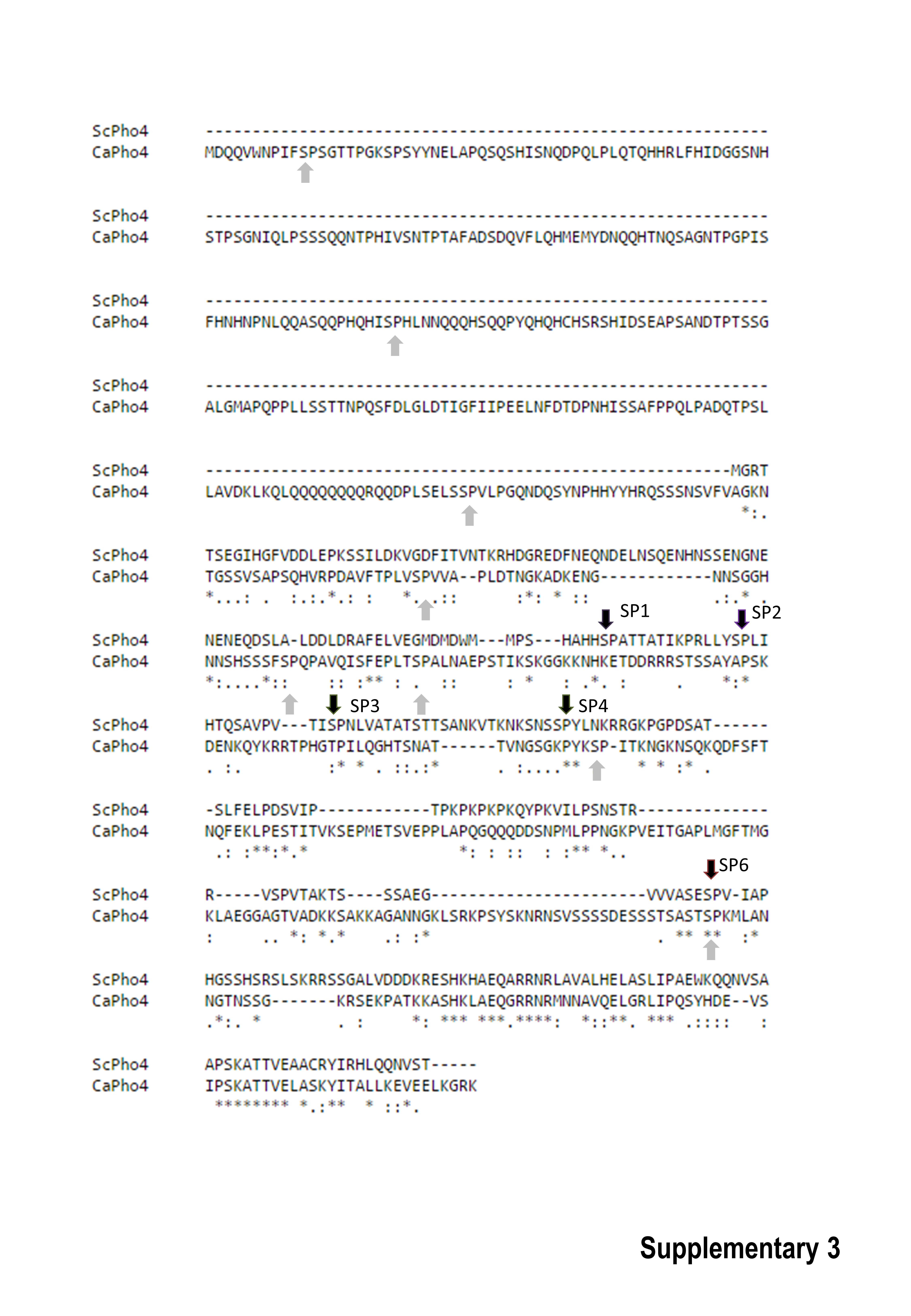

Supplement: Supplementary Figure 3 — Comparison of ScPho4 and CaPho4 sequences. ScPho4 and CaPho4 were aligned using the default setting of Clustaw2. Dark arrows sign ScPho4 reported phosphorylation sites (Sp1, SP2, SP3, SP4, and SP6) while gray arrows indicate putative phosphorylation sites detected in CaPho4 sequence. [file Image3.TIF]

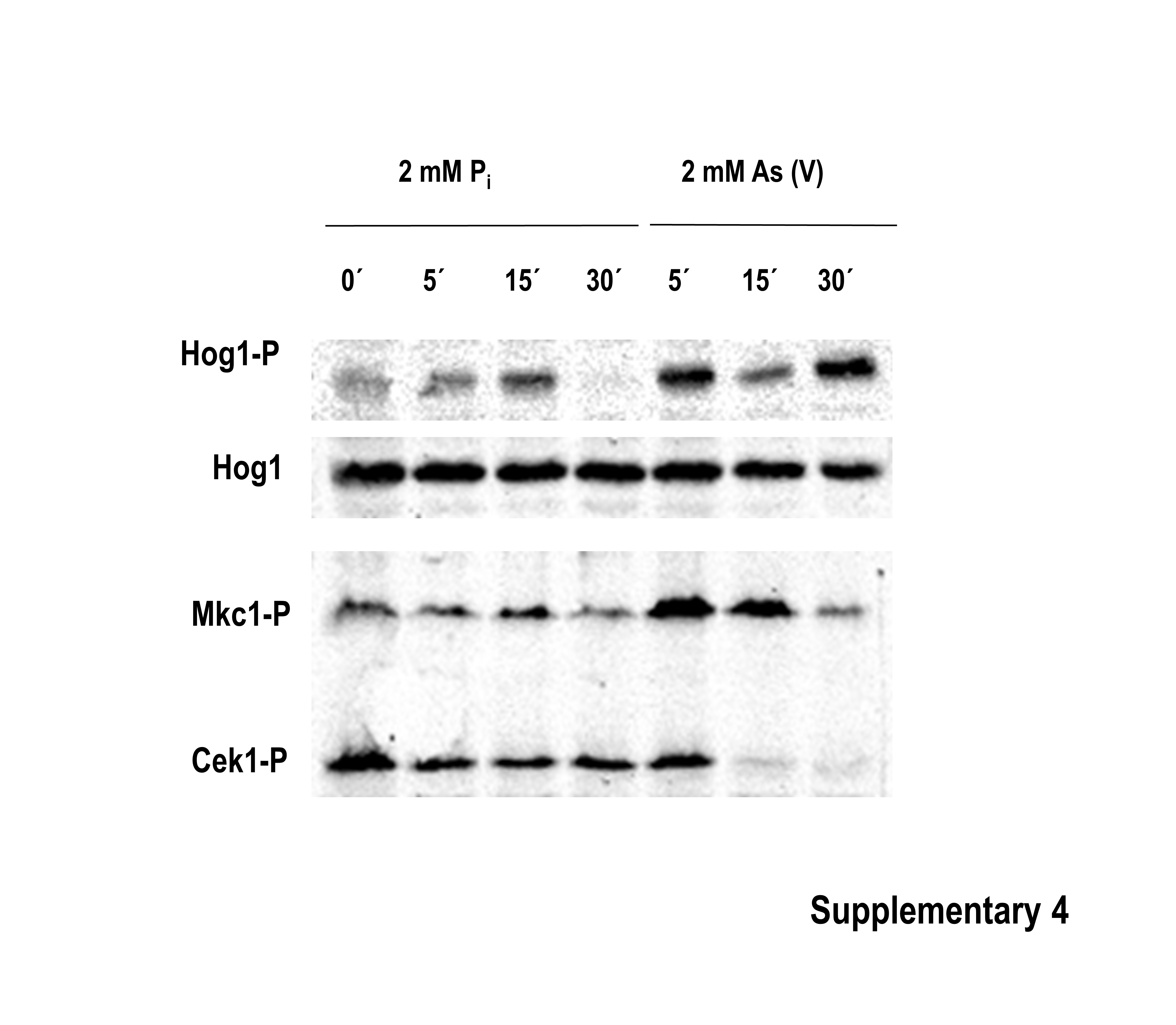

Supplement: Supplementary Figure 4 — Pi and As (V) trigger different signaling pathways. A wild type strain (CAF 2) grown exponentially in SD medium was split in two and 2 mM Pi or 2 mM As (V) was added to each culture. Samples were taken at different time points and MAP kinase phosphorylation was analyzed using specific antibodies. Hog1-P, Cek1-P, and Mkc1-P designate the phosphorylated form of the MAPKs while Hog1 indicates the total Hog1 protein. [file Image4.TIF]

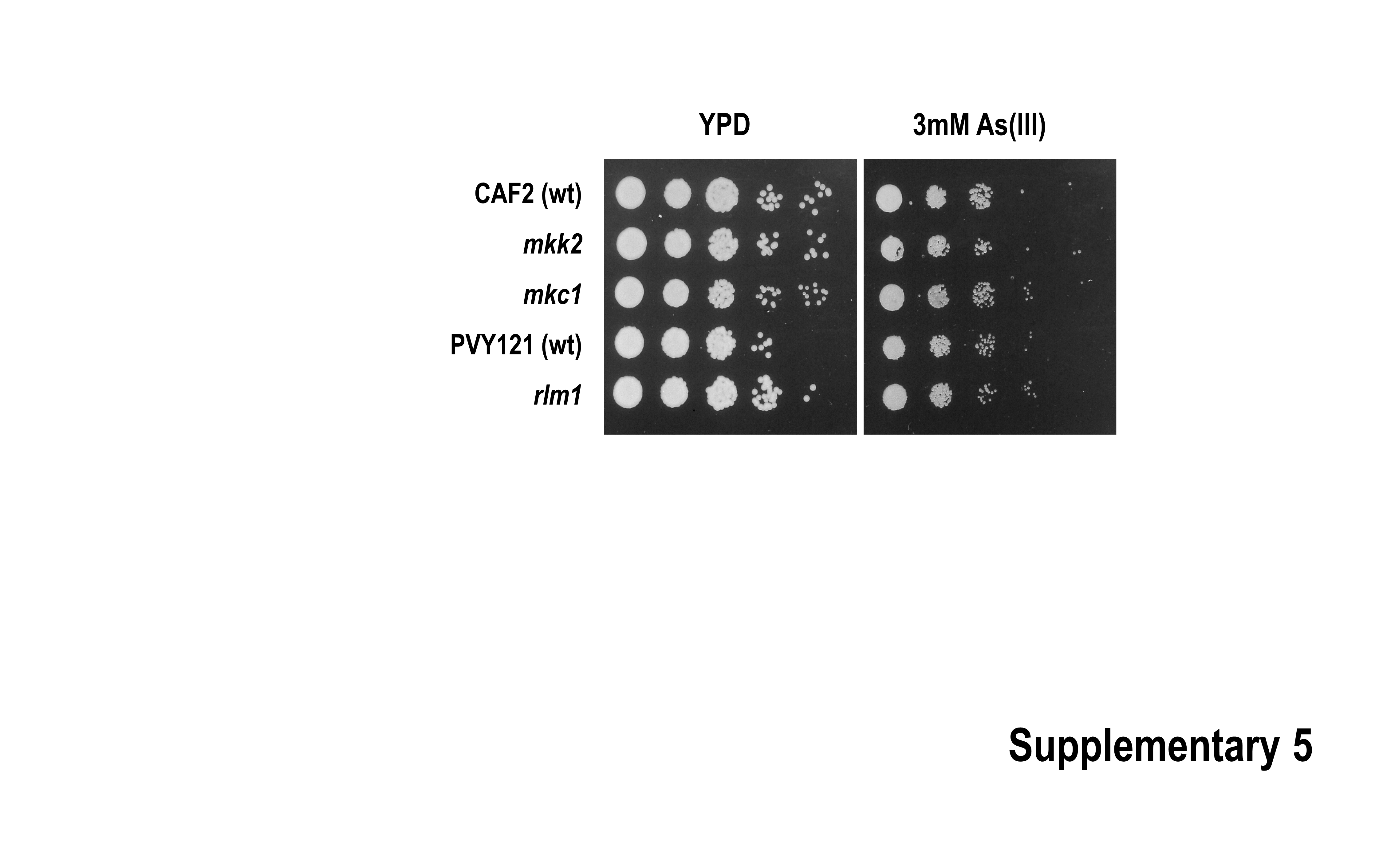

Supplement: Supplementary Figure 5 — Defective mutants in the CWI pathway are not susceptible to As (III). Ten-fold dilutions of the indicated strains were spotted on YPD and YPD plus 3 mM As (III). Plates were incubated at 37°C for 24 h. [file Image5.TIF]
